# Supplementary figures and images for: Proximate determinants of the frequency of mosquito sounds: separating species-specific effects from environmentally driven variations - Implications for AI species recognition
Source: PLoS One. 2026 Mar 4;21(3):e0343060. doi: 10.1371/journal.pone.0343060 (PMC12959652; doi:10.1371/journal.pone.0343060)

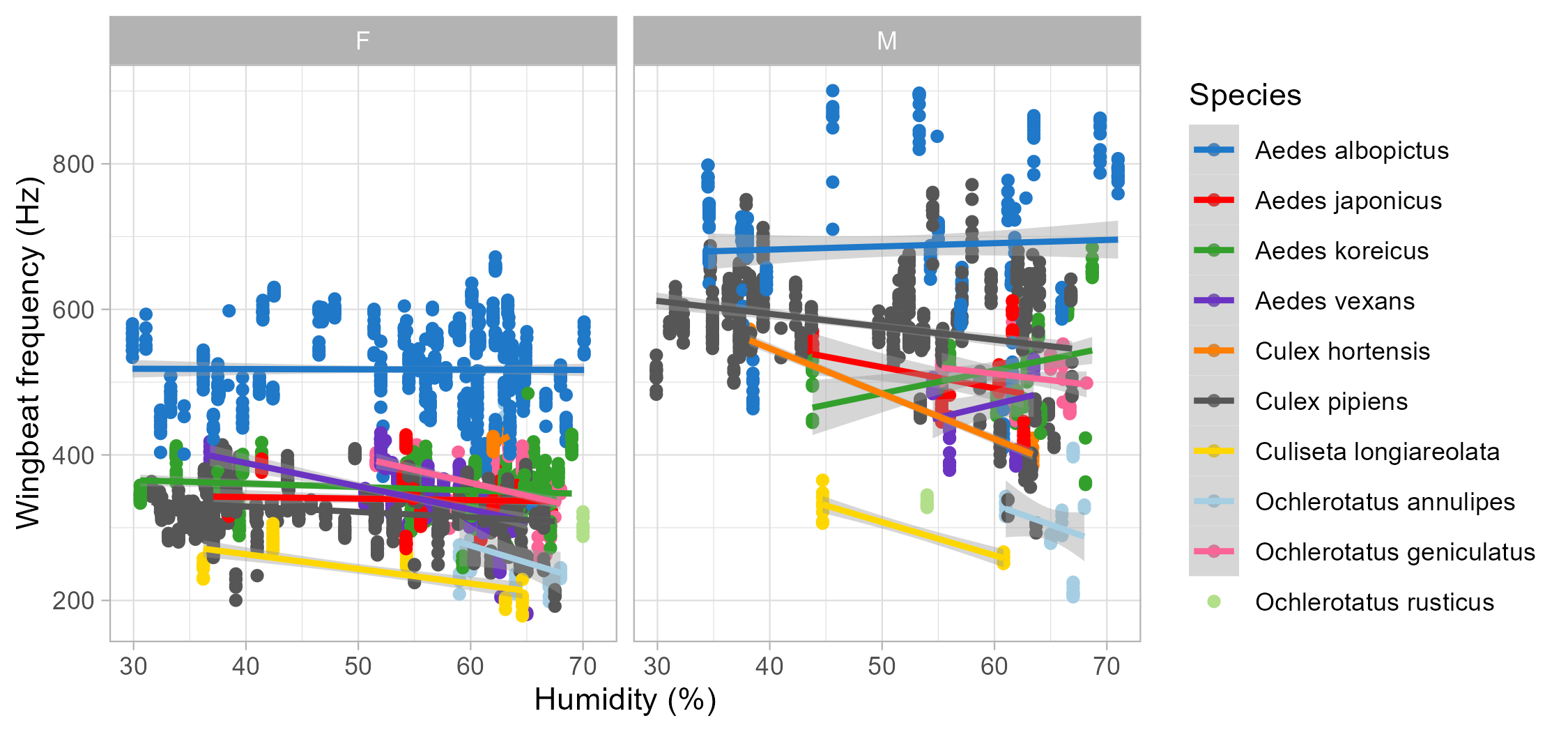

Supplement: S1 Fig — F = females, M = males. (TIFF) [file pone.0343060.s006.tiff]

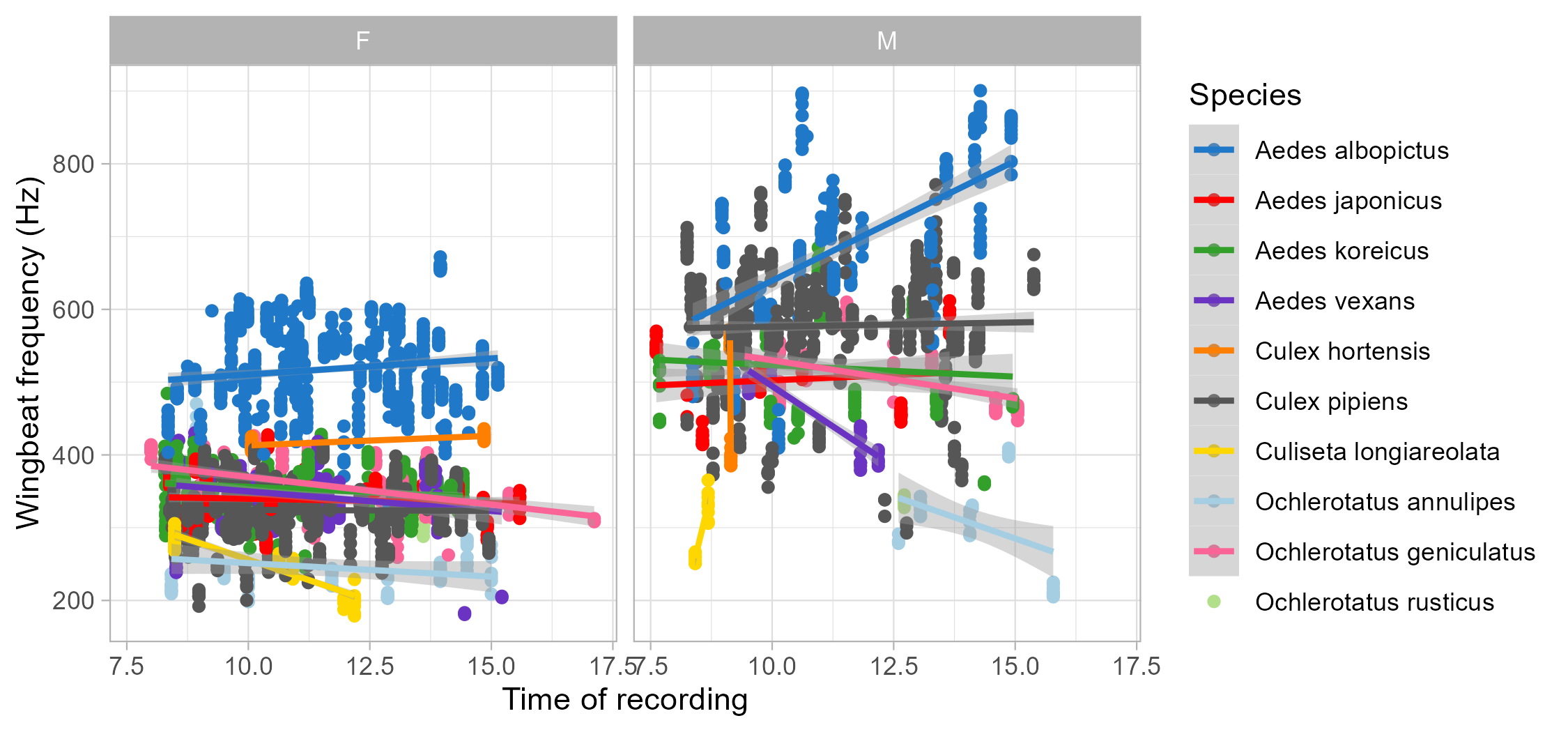

Supplement: S2 Fig — F = females, M = males. (TIFF) [file pone.0343060.s007.tiff]

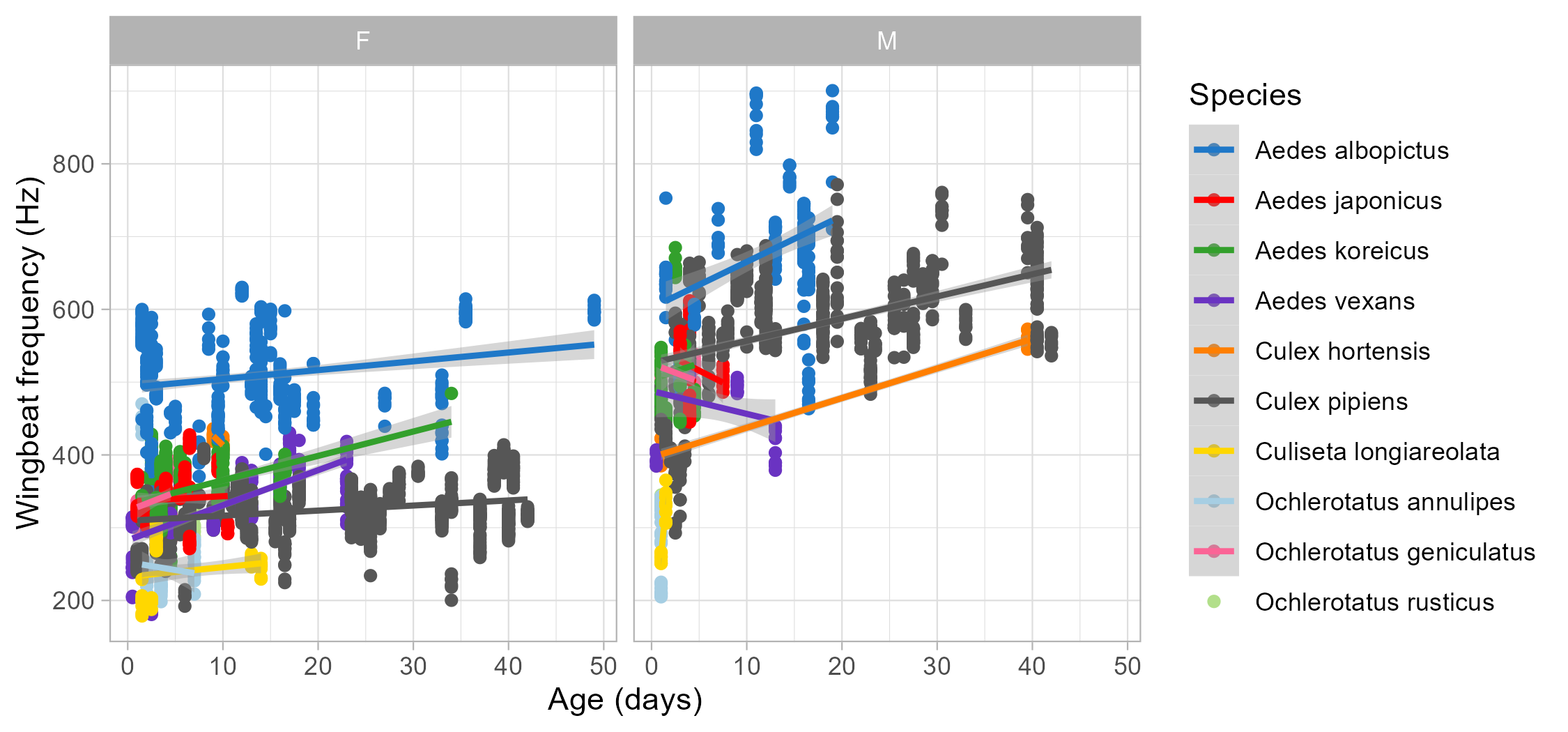

Supplement: S3 Fig — F = females, M = males. (TIFF) [file pone.0343060.s008.tiff]

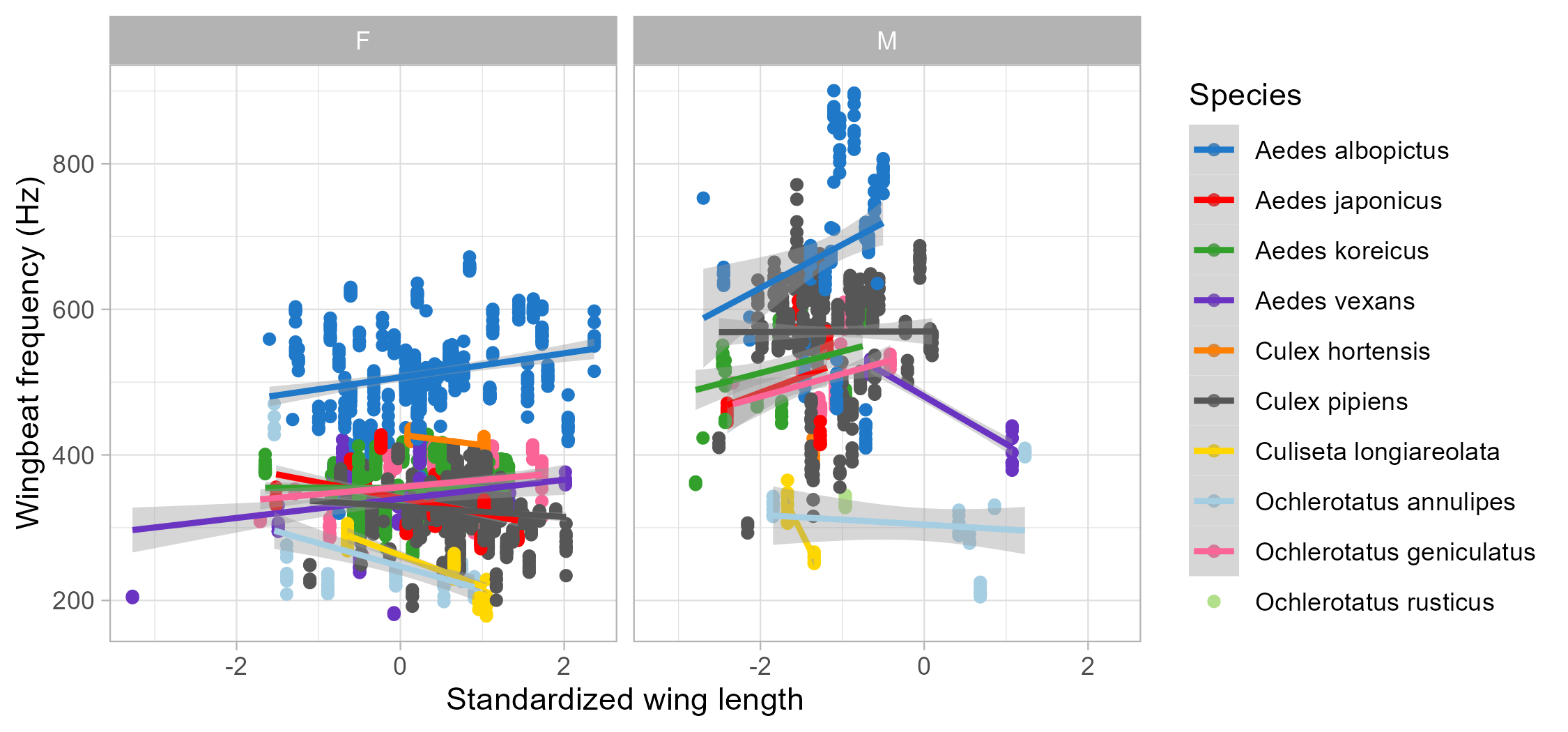

Supplement: S4 Fig — F = females, M = males. (TIFF) [file pone.0343060.s009.tiff]

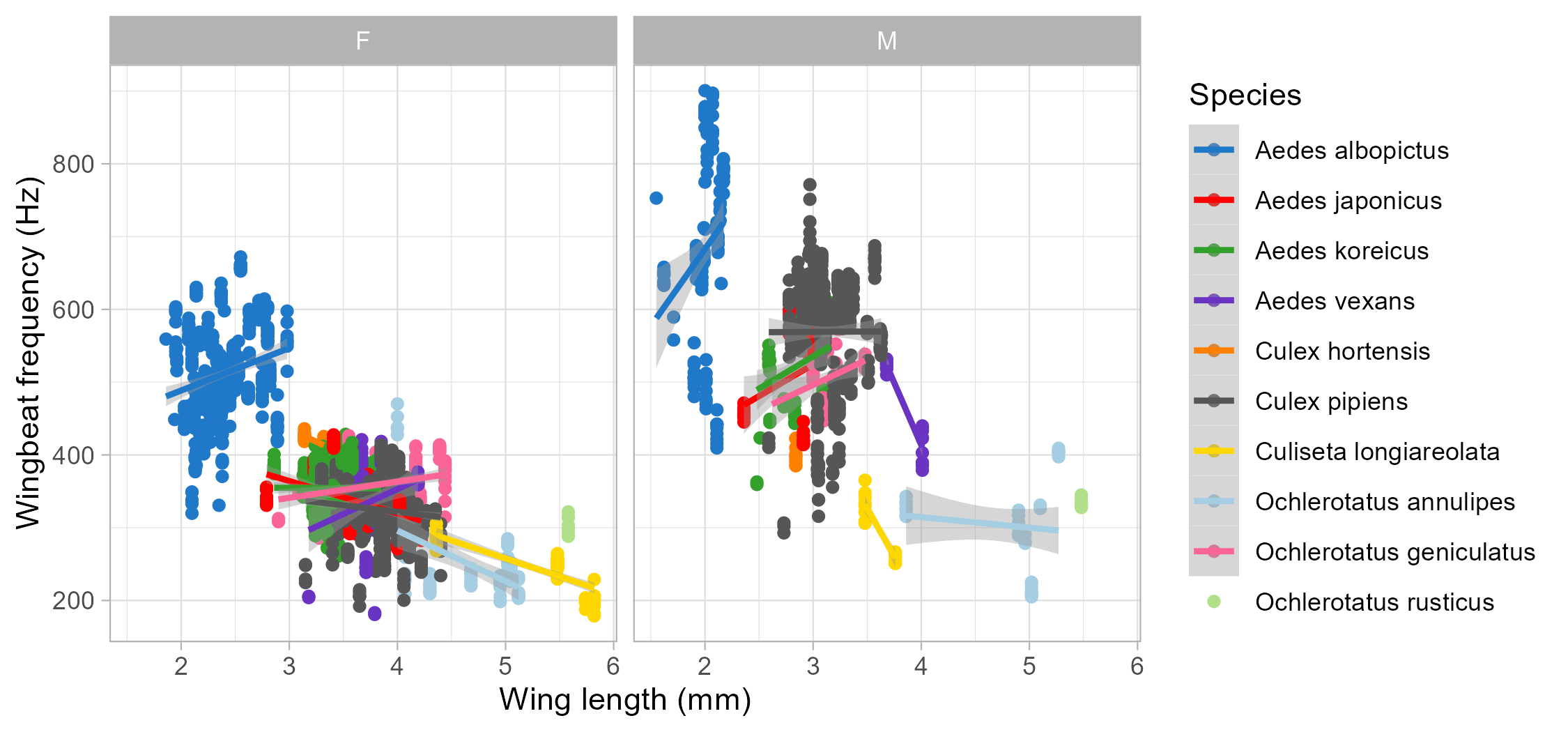

Supplement: S5 Fig — F = females, M = males. (TIFF) [file pone.0343060.s010.tiff]
