# Supplementary material for: Proximate determinants of the frequency of mosquito sounds: separating species-specific effects from environmentally driven variations - Implications for AI species recognition
Source: PLoS One. 2026 Mar 4;21(3):e0343060. doi: 10.1371/journal.pone.0343060 (PMC12959652; doi:10.1371/journal.pone.0343060)
Supplement: Attachment — Submitted filename: review.pdf [file pone.0343060.s012.pdf]

The authors have make significant efforts to answer all my questions and I think the study worth being published, provided on adding the information given below on comment 3).

FOLLOWING-UP ON PREVIOUS COMMENTS

3) “The reduction of external sound was not as good as when the soundproof box was fully closed, but it was much better than no soundproof box. This was added in the methods, lines 206-209”

—> I cannot read this important information in lines 206-209

12) Is there a particular reason why Villarreal et al. 2017 is not cited in the litterature review for effect of temperature?

NEW COMMENTS

14) “There was a strong variability in willingness to fly between individuals, with some mosquitoes flying non-stop throughout the 10min period, and some flying only for a second when stimulated. We included all mosquitoes that were observed flying in the analysis, regardless of their flight duration during the trial.”

—> If available, it could be interesting to have statistics on the duration of fly, and whether it is species specific, to check if circadium-dependant factors can affect this, e.g. whether night species only had short flights etc

15) the cage is 10x10x10 cm3. Could this small dimension have an effect on the mosquito flight? Could it be a limitation of the study as compared to natural conditions?
