# Supplementary material for: Proximate determinants of the frequency of mosquito sounds: separating species-specific effects from environmentally driven variations - Implications for AI species recognition
Source: PLoS One. 2026 Mar 4;21(3):e0343060. doi: 10.1371/journal.pone.0343060 (PMC12959652; doi:10.1371/journal.pone.0343060)
Supplement: Attachment — Submitted filename: Response to reviewers.docx [file pone.0343060.s013.docx]

We thank the academic editor and reviewers for their interest in the manuscript and their comments. Please find below our responses to the comments. The lines indicated in this document correspond to the lines in the new version of the manuscript without track changes (“Manuscript”).

Academic Editor

Journal Requirements:

When submitting your revision, we need you to address these additional requirements.

1. Please ensure that your manuscript meets PLOS ONE's style requirements, including those for file naming. The PLOS ONE style templates can be found at

<https://journals.plos.org/plosone/s/file?id=wjVg/PLOSOne_formatting_sample_main_body.pdf> and

[https://journals.plos.org/plosone/s/file?id=ba62/PLOSOne_formatting_sample_title_authors_affiliations.pdf](https://track.editorialmanager.com/CL0/https:%2F%2Fjournals.plos.org%2Fplosone%2Fs%2Ffile%3Fid=ba62%2FPLOSOne_formatting_sample_title_authors_affiliations.pdf/1/010f019a4e53717a-2528c147-25d6-4e78-a2cb-6ae7c0098bd8-000000/i0XO3oODTr36LA-Ghlgl-FYJcnY6O_eI48LCjPkoNX4=235)

The manuscript’s format has been modified according to the guidelines (heading, figures, tables…).

2. Please note that PLOS One has specific guidelines on code sharing for submissions in which author-generated code underpins the findings in the manuscript. In these cases, we expect all author-generated code to be made available without restrictions upon publication of the work. Please review our guidelines at <https://journals.plos.org/plosone/s/materials-and-software-sharing#loc-sharing-code> and ensure that your code is shared in a way that follows best practice and facilitates reproducibility and reuse.

The codes have been publicly shared either in a separate repository or in the figshare repository that also contains the data:

- Automatic detection of mosquito sounds: <https://github.com/MosquiTUNE/Mosquito_sound_detection_methods/tree/main>
- Sound measurements: https://figshare.com/s/084db2848ed6bad49a2e
- Statistical analysis: https://figshare.com/s/084db2848ed6bad49a2e

3. In your Methods section, please provide additional information regarding the permits you obtained for the work. Please ensure you have included the full name of the authority that approved the field site access and, if no permits were required, a brief statement explaining why.

Because we only collected invertebrates, did not sample in private or protected areas or protected species, no permits were required. This was added in the methods, lines 176-179.

4. Thank you for uploading your study's underlying data set. Unfortunately, the repository you have noted in your Data Availability statement does not qualify as an acceptable data repository according to PLOS's standards.

At this time, please upload the minimal data set necessary to replicate your study's findings to a stable, public repository (such as figshare or Dryad) and provide us with the relevant URLs, DOIs, or accession numbers that may be used to access these data. For a list of recommended repositories and additional information on PLOS standards for data deposition, please see [https://journals.plos.org/plosone/s/recommended-repositories](https://track.editorialmanager.com/CL0/https:%2F%2Fjournals.plos.org%2Fplosone%2Fs%2Frecommended-repositories/1/010f019a4e53717a-2528c147-25d6-4e78-a2cb-6ae7c0098bd8-000000/I4aAMNtj4805IZjRRYNaNQxydiZ67GNFFNgEGEEmOYQ=235).

The data was deposited on Figshare, and the data availability statement has been modified.

DOI: 10.6084/m9.figshare.30230581

Private link: https://figshare.com/s/084db2848ed6bad49a2e

5. When completing the data availability statement of the submission form, you indicated that you will make your data available on acceptance. We strongly recommend all authors decide on a data sharing plan before acceptance, as the process can be lengthy and hold up publication timelines. Please note that, though access restrictions are acceptable now, your entire data will need to be made freely accessible if your manuscript is accepted for publication. This policy applies to all data except where public deposition would breach compliance with the protocol approved by your research ethics board. If you are unable to adhere to our open data policy, please kindly revise your statement to explain your reasoning and we will seek the editor's input on an exemption. Please be assured that, once you have provided your new statement, the assessment of your exemption will not hold up the peer review process.

The data availability statement has been updated with reference to the data repository on figshare.

6. Please include your full ethics statement in the ‘Methods’ section of your manuscript file. In your statement, please include the full name of the IRB or ethics committee who approved or waived your study, as well as whether or not you obtained informed written or verbal consent. If consent was waived for your study, please include this information in your statement as well.

The ethics statement was included in the methods section of the manuscript, lines 176-179

7. We note that Figure 1 in your submission contain map image which may be copyrighted. All PLOS content is published under the Creative Commons Attribution License (CC BY 4.0), which means that the manuscript, images, and Supporting Information files will be freely available online, and any third party is permitted to access, download, copy, distribute, and use these materials in any way, even commercially, with proper attribution. For these reasons, we cannot publish previously copyrighted maps or satellite images created using proprietary data, such as Google software (Google Maps, Street View, and Earth). For more information, see our copyright guidelines: http://journals.plos.org/plosone/s/licenses-and-copyright.

We require you to either (1) present written permission from the copyright holder to publish these figures specifically under the CC BY 4.0 license, or (2) remove the figures from your submission:

a. You may seek permission from the original copyright holder of Figure 1 to publish the content specifically under the CC BY 4.0 license.

We recommend that you contact the original copyright holder with the Content Permission Form (http://journals.plos.org/plosone/s/file?id=7c09/content-permission-form.pdf) and the following text:
“I request permission for the open-access journal PLOS ONE to publish XXX under the Creative Commons Attribution License (CCAL) CC BY 4.0 (http://creativecommons.org/licenses/by/4.0/). Please be aware that this license allows unrestricted use and distribution, even commercially, by third parties. Please reply and provide explicit written permission to publish XXX under a CC BY license and complete the attached form.”

Please upload the completed Content Permission Form or other proof of granted permissions as an "Other" file with your submission.

In the figure caption of the copyrighted figure, please include the following text: “Reprinted from [ref] under a CC BY license, with permission from [name of publisher], original copyright [original copyright year].”

b. If you are unable to obtain permission from the original copyright holder to publish these figures under the CC BY 4.0 license or if the copyright holder’s requirements are incompatible with the CC BY 4.0 license, please either i) remove the figure or ii) supply a replacement figure that complies with the CC BY 4.0 license. Please check copyright information on all replacement figures and update the figure caption with source information. If applicable, please specify in the figure caption text when a figure is similar but not identical to the original image and is therefore for illustrative purposes only.
The following resources for replacing copyrighted map figures may be helpful:

USGS National Map Viewer (public domain): http://viewer.nationalmap.gov/viewer/
The Gateway to Astronaut Photography of Earth (public domain): http://eol.jsc.nasa.gov/sseop/clickmap/
Maps at the CIA (public domain): https://www.cia.gov/library/publications/the-world-factbook/index.html and https://www.cia.gov/library/publications/cia-maps-publications/index.html
NASA Earth Observatory (public domain): http://earthobservatory.nasa.gov/
Landsat: http://landsat.visibleearth.nasa.gov/
USGS EROS (Earth Resources Observatory and Science (EROS) Center) (public domain): http://eros.usgs.gov/#
Natural Earth (public domain): <http://www.naturalearthdata.com/>

The map has been removed, and the collection data added as supplementary material (S5 Table). Figures and tables numbers and their references in the text have been adjusted accordingly.

8. We are unable to open your Supporting Information file [File Name]. Please kindly revise as necessary and re-upload.

The supporting information files have been revised and re-uploaded.

9. If the reviewer comments include a recommendation to cite specific previously published works, please review and evaluate these publications to determine whether they are relevant and should be cited. There is no requirement to cite these works unless the editor has indicated otherwise.

The specific published work that was mentioned by the reviewers was relevant and often already cited elsewhere in the manuscript.

**Reviewer #1**: The manuscript “Environmental and biotic determinants of mosquito wingbeat frequency and implications for AI-based species recognition” is a timely and important study that addresses a central limitation in current mosquito acoustic monitoring: the lack of integration of environmental and biotic variability into species classification frameworks. The manuscript presents an impressive dataset (475 individuals from 15 species) and provides novel quantitative insights into within- and between-species repeatability of wingbeat frequency. The work is rigorous, relevant, and provides clear perspectives for future research, particularly regarding how biological and environmental covariates could improve AI-based classification of vector species.
The manuscript is scientifically strong and has clear potential for publication in PLOS ONE. However, revisions are necessary to improve clarity for a general audience, ensure methodological transparency, and align statements across sections. Once these issues are addressed, the paper will make an important contribution to bioacoustics and vector ecology by quantifying the influence of environmental and biotic variability on mosquito sounds and demonstrating its relevance for AI-based species recognition.

Strengths:
- Addresses a relevant and emerging intersection between bioacoustics and vector surveillance.
- Employs a well-chosen mixed-model framework with clear variance partitioning.
- Includes non-model mosquito species, expanding the taxonomic and ecological scope of acoustic studies.
- Provides actionable implications for improving AI-based mosquito recognition systems.

Thank you for the appreciation.

Specific Comments

1. Accessibility and scope of the Introduction

The Introduction is scientifically rich but currently too narrowly focused for the journal’s broad audience, i.e. briefly explain repeatability (statistical proportion of variance due to consistent among-individual or among-species differences) and why it matters for classification robustness and introduce vector species in simple terms (mosquitoes capable of transmitting pathogens).

Repeatability and its importance for classification system was explained more in the last paragraph of the introduction, lines 138-144.

In addition, a sentence has been added in the beginning of the introduction to define mosquitoes as vector of pathogens, lines 43-45.

Lines 41–70: Reorganize to introduce the ecological and applied relevance of mosquito acoustic monitoring before diving into technical details. For a general audience, it would help to first explain why acoustic species identification matters (e.g., vector surveillance & disease control).

A sentence has been added to the beginning of the introduction to highlight the importance of mosquito monitoring, lines 45-47.

In addition, the importance of species identification has been added, lines 48-53.

Line 45: Briefly explain opto-acoustics for non-specialists

A brief explanation of the different type of data, including the opto-acoustics, has been added in the appropriate section, lines 58-62

Lines 49–51: If they reach accuracy of 97% what is there to improve? And what is meant by underutilized and why is that important in this context? Clarify whether these results apply only under controlled laboratory conditions and why field deployment remains limited (e.g., lack of robustness, difficulty in implementation, or lack of trust). Strengthen the logical transition to “However…” in line 51.

This section has been expanded to explain the conditions of high accuracy and why they are not yet deployed in the field, lines 66-75.

Line 64: Quantify “data remain scarce”—for example, indicate the approximate number of studies or species covered (“data currently exist for ~6 studies on 2–3 model species”).

The sentence has been modified as suggested, lines 91-92

Tables 1 & 2: These tables are rich but create an impression that the topic is well explored, which contradicts the statement of scarcity. Consider moving them later in the Introduction or summarizing them graphically. A visual summary (e.g., species icons with circles representing investigated factors) would help illustrate the strong research bias toward Aedes aegypti.

The text has been modified to highlight that the scarcity refers mostly to the assessment of impact of environmental factors, lines 89-91

Figures were created as suggested to replace the tables. The placement of Fig 1 and Fig 2 has been modified accordingly, to fit with the order they appear in the text. The titles and captions have also been modified. Subsequent tables and figures number in the text have been adjusted accordingly.

The original tables have been included as supplementary material (S1 Table and S2 Table) for readers that are interested in more of the specific effects.

2. Clarify the rationale for the focus on Aedes aegypti

The Introduction would benefit from a short statement explaining why Aedes aegypti dominates the literature (e.g., ease of laboratory maintenance, relevance as a disease vector). This contextualization will help readers understand the novelty of including less-studied species.

A short text has been added after the tables to explain the importance of the three most studied species, in particular Aedes aegypti, lines 103-106.

3. Methods

While the methods are thorough, several key steps need clarification to ensure reproducibility and transparency.

Line 127: Rephrase for clarity: “Wild-caught mosquitoes were recorded shortly after capture. Individuals reared from laboratory colonies did not differ in wingbeat frequency and were therefore included in the analysis.”

The text was modified as suggested, except for the first sentence that was left out because we did not record wild-caught adult mosquitoes, lines 162-164.

Lines 148–150: Clarify how acoustic stimulation works within a soundproof box. Does the operator reach into the box or use an external tool?

The protocol for the stimulation was clarified, lines 206-209.

Table 3 (Age): Clarify that the age range reflects individuals bred from wild-collected eggs or larvae, not adult field captures. Currently it is unclear whether wild adults were recorded.

The age reflects the number of days since adult emergence. It was clarified in the Table. In addition, the text was clarified in the methods to indicate that no field-caught adults were recorded, lines 162-164

Lines 169–174: Move this paragraph earlier (before recordings) and expand on why estimating median age per cage is a reasonable approach.

The paragraph was moved earlier (lines 135-158), and an explanation has been added on the relevance of using the median in this situation, lines 183-190.

Line 177: Specify the software environment of the “custom-made mosquito detection module” (e.g., R, Python, MATLAB). Indicate whether the code is publicly available and provide a repository link. If not, explain why.

The software environment was added to the description. In addition, the code was uploaded into a public repository, and its link has been added to the paragraph, lines 241-242

Line 190: Explain selection criteria more explicitly—what was done when only one high-quality sound was available? Did usable sounds cluster temporally (e.g., early vs. late in recording)?

This section has been expanded a little to explain in more details the selection process, lines 254-256.

Usable sounds did not cluster temporally and were distributed evenly throughout the recordings. Recordings were 10 minutes, so 600s, and the average timestamp of the sounds was 289 ± 175 s, meaning that there was a bit more sounds selected in the first half of the recording than in the second half, but the trend is small and should not generate bias in our analysis. Based on the sound distribution plot, it seems we had a small bias in selecting the sounds at the very beginning of the recording during the manual verification step, but overall, the usable sound distribution is pretty uniform within all recordings.

The text has been modified to reflect that the sounds were indeed evenly distributed within the recordings, lines 255-256.

Line 192: Again, specify if analysis code is available.

The script has been included into the figshare repository with the data, and the link added to the text, lines 257

Line 196: Explicitly name the extracted output parameter (e.g., “f1_freq = mean frequency of the first harmonic”).

The text has been modified to name the extracted output parameter, lines 262

Line 209: Explain what “different hierarchical levels” refers to (sounds within individuals, individuals within species) and why repeatability is the metric of interest. Define its possible range (0 = no consistency; 1 = complete consistency).

The text has been modified to better describe the different levels of repeatability reflecting the hierarchical structure of data, lines 273-280. Because the possible range was already mentioned in the introduction following another comment, it was not repeated here.

Line 211: Describe the specific parametric bootstrap procedure used (number of simulations, software function). If the scripts are not shared, reproducibility is limited—consider providing them as supplementary material.

The script has been added to the figshare repository: https://figshare.com/s/084db2848ed6bad49a2e

The number of simulations was added in the text, line 280

Line 232: Adjust the Abstract to clarify that analyses were conducted on 10 species, though recordings were made for 15.

A line was added to the abstract to clarify this aspect, lines 28-29.

Table 4: Specify whether numbers represent all recorded individuals or those included in analyses.

The specification was added in the caption of the table, lines 306-307

Table 5: Add units (Hz), and consider presenting range and median in addition to mean ± SD. High SD values (often >10 % of the mean) merit brief comment.

The units, median and range have been added to the table, and the caption modified accordingly. In addition, a comment was added regarding the high standard deviation values, lines 326-329.

Table 6: Add a short note in the Introduction explaining what repeatability values represent and how to interpret them (e.g., “values near 1 indicate high consistency across individuals or species”).

In complement to a previous comment and text modification, an explanation was added in the introduction about what repeatability values represent and how to interpret them, lines 138-145

Line 269: Clarify why wing length was standardized within species and discuss potential implications of this choice on the non-significant result.

Wing length is intrinsically linked to the species (i.e. some species have larger wings than the other), and species-specific effects would drive by default a large part of the variance in wingbeat frequency. We decided to standardize the variable in order to estimate effects that are independent of the species-specific signals (that are already included in the model as random effect).

When wing length was not standardized, it did become a significant predictor of wingbeat frequency, but due to its intrinsic relationship to the species variable, this significant relationship should reflect species-specific effects and not effect caused by interspecific variance in wing-length. Therefore, we preferred to use the standardized version, especially since the species/wing length/wingbeat frequency relationship has already been established in several papers.

The text was modified both in the methods, lines 232-237, and the discussion, lines 441-444

Lines 270–276 / Fig. 2: Provide a clearer description of random-slope results. Highlight that not all species respond similarly to temperature, and discuss how unequal sample sizes per species may influence slope estimates.

The text has been modified to better describe the random slope results and the impact of the sample size, lines 368-373.

Line 284: Spell out “a good number of non-model species (n = 10 analyzed)” to emphasize novelty.

The sentence was modified according to the recommendation line 376

4. Results and Discussion

The Results are generally clear and statistically well presented, but some interpretation could be expanded for accessibility:

- Consider briefly summarizing expected vs. observed repeatability patterns (Table 6) for readers unfamiliar with the metric.

The text has been modified to better explain the differences in repeatability and their biological relevance, lines 331-342

- The Discussion effectively highlights temperature and sex as key predictors but could benefit from a clearer paragraph summarizing the practical implications for AI-based classification (e.g., integrating temperature correction factors or including metadata in training).

A paragraph discussing these practical implications was already present later in the discussion, and has been modified to highlight the role of temperature, lines 490-494.

- The final section on mate selection and individual quality is interesting but peripheral. Condense or explicitly link it to the study’s broader message about acoustic variability and consistency.

The text of this section has been condensed.

Reviewer #2: The authors present a study of the variation of mosquitoes' wingbeat frequency (WBF) accross species, sex, temperature and other environmental conditions. I found the study is intesresting because too many studies does not take into consideration the context where the mosquito WBF are recorded.

MAJOR COMMENTS

1) insufficient information on the "time of recording" parameter
Line 104-106, 181 : "due to the strong circadian variations in mosquitoes activity, we evaluated the impact of the time of recording"
--> no methodological informaiton on this parameter, i.e. how time of recording was controlled and which ranges it took. What time were the mosquitoes recorded in the day and when was it as compared to the mosquito circadium time.
Did you record mosquitoes at a time independnat of wether they were day or night mosquitoes?

This is a good point. A more detailed description of the time of recording has been added in the methods, lines 217-221.

Further explanations and discussion of this limitation have been added in the discussion, lines 458-463

This aspect has also been included into Table 3 (previously Table 5) caption.

2) Insufficient information on acoustic processing
Line 148 / 181: not clear how the authors dealt with superimposed WBF when more than 1 mosquito was flying at a time

There was only one mosquito flying at a time. The methods were modified to better describe this aspect, lines 192-209.

3) Insufficient information on acoustic recording
Line 148 / 181: not clear how the authors proceed to record the mosquitoes. More information is needed to know the behaviour of the mosquitoes during the recording, e.g.:
How was managed the opening of the soundproof box to make them fly and then record them? Did the authors wait for some time after the sonudproof box was closed?
How long time do the mosquito fly in the cage in general?
What are the mean duration of an long enough flight?
Was it full darkness in the soundproof chamber? If yes, what would be the effect on WBF / mosquito behaviour?

There was an opening on the side of the soundproof box which remained open and allowed us to interact with the cage. The reduction of external sound was not as good as when the soundproof box was fully closed, but it was much better than no soundproof box.

This was added in the methods, lines 206-209

The mosquito flying sound was not always picked up by the microphones if the mosquito was too far, so we cannot know precisely how long they were usually flying. However, when running the experiments, it was obvious that the motivation to fly varied largely from one individual to the next. The mosquitoes were recorded for 10 minutes and flew anywhere from one second when stimulated to the full 10 minutes without stopping. We considered that any flight was a long enough flight, even the shortest one, but we established a threshold at the level of the sound, with a long-enough sound being at least 0.2s (as indicated line 251). This aspect was also added to the methods, lines 209-212

It was not full darkness in the soundproof box, the interior box was lit, this was added to the text, lines 195-196

4) Another parameters that affects male WBF is the acoustic detection of the female (table 2, ...):
See the review book chapter you cite (49): male fundamental WBFs are observed to reach up to 1 000 Hz for short periods of time in swarms (Pantoja-Sanchez et al. (2019), Garcia Castillo et al., 2021; <https://doi.org/10.1242/jeb.243535> , <https://doi.org/10.1126/sciadv.abl4844>). Then, the behaviour could also be a factor of WBF.

While we previously included the swarming status, it is true that the acoustic detection of the female further affects the wingbeat frequency. The Pantoja Sanchez and Garcia-Castillo references were added to the table in the column of the swarming status. The modification of WBF in response to the sound of a sexual partner, same-sex conspecifics and pure tones was also added in the table (now S1 Table) and the figure created from it based on reviewer 1 comments (Fig 1).

MINOR COMMENTS

5) line 78 "mating does not appear to alter wingbeat frequency". WBF is altered during mating I guess you meant "mated status"?

Yes, we did mean mating status. The sentence was modified, lines 114-115.

6) Line 140: "N=12" : not clear if it is the number of mosquitos used per cage.

The “N=12” refers to the number of individuals that appeared to be injured after the transfer into the cage, and were removed from the experiment. Only one mosquito was recorded at a time per cage; the paragraph was modified to make this clearer, as previously mentioned, lines 192-201

7) Table 3: Usually, good temperature sensors have a precision of ~1°C, then having 1 digit precision in table 3 can be seen as an excess of precision: what is the tempreature accuracy of Voltcraft DL-210TH? Also it seems that Voltcraft DL-210TH is a datalogguer, not a sensor (not checked carefully). In this case, you should say which sensor was plugged to the data logguer and its temperature accurary.
Humidity sensor are even far less precise than temperature sensor and a 1 digit precision is not meaningfull.

The Voltcraft DL-210TH is described as a logger but it contains internal sensors and directly measures the temperature and humidity. This precision was added in the text, lines 216-217.

After verification, its temperature accuracy is indicated as +/- 0,5 °C. Therefore, the digits for temperature have been rounded to the nearest 0.5 digit, and the 1-digit precision of the humidity values were removed.

8) Table 5, line 320: 2-digits for WBF are useless and give a wrong sense of precision which does not exist on . I would advice not to put any digits, and at least to remove the 2nd digit.

The 2-digits were removed from the WBF values to better indicate the real precision of the measurement.

9) line 300: and 302: please gives the amplitude of change in Hz/°C and statistical test results; Discuss wether it could make any difference.

The amplitude of change in Hz/°C was indicated for each species/sex in the new Table 5 (lines 359-360). The results of the statistical test are indicated in the result section of the manuscript, lines 355-356. The results and discussion have been modified to discuss these results in the appropriate sections, lines 369-374 and 415-431.

We are not sure what the “discuss whether it could make any difference” refers to. If the previous section is not sufficient, please provide further detail on how we could expand on the topic.

10) Given a species, are the slopes the same between males and females? This could be interesting in terms of mosquito hearing; indeed, males hear the difference frequency between they own WBF and that of the nearby female (distortion product from their antennae; see Warren et al 2009) ; this would tell us whether their hearing organ tuning would need to have the same temperature gradiant for instance. Indeed, hearing difference-tones cancels the effect of temperature when hearing each other, as suggested by Lapshin and Vorontsov (2017).

The slopes are generally not the same between males and females, even if the scale of the difference varies between species. The hearing and recognition aspect has been added to the discussion, lines 421-431

11) line 388: the harmonic convergence theory is somewhow out of date (e.g. see the review chapter Feugere et al 2022; Somers et al 2022; Warren et al. (2009))

In response to this comment and reviewer 1’s suggestion to reduce this section of the discussion, all mentions to harmonic convergence have been removed from the discussion.

12) see also Villarreal et al., 2017 for effect of temperature

The Villareal paper was further cited as a source for the calculation of the WBF/temperature slope, lines 359-360

13) In the abstract, I would not highlight that female's WBF are lower than males' one, as it is really well established.

This part of the sentence was removed in the abstract.

NB: I have not checked the data on figshare
