# Supplementary material for: Proximate determinants of the frequency of mosquito sounds: separating species-specific effects from environmentally driven variations - Implications for AI species recognition
Source: PLoS One. 2026 Mar 4;21(3):e0343060. doi: 10.1371/journal.pone.0343060 (PMC12959652; doi:10.1371/journal.pone.0343060)
Supplement: Attachment — Submitted filename: Response_to_reviewers_auresp_2.docx [file pone.0343060.s014.docx]

The authors have make significant efforts to answer all my questions and I think the study worth being published, provided on adding the information given below on comment 3).

FOLLOWING-UP ON PREVIOUS COMMENTS

3) “The reduction of external sound was not as good as when the soundproof box was fully closed, but it was much better than no soundproof box. This was added in the methods, lines 206-209”

—> I cannot read this important information in lines 206-209

You are right, sorry about this oversight. A sentence addressing the box was added in the manuscript lines 210-211.

12) Is there a particular reason why Villarreal et al. 2017 is not cited in the litterature review for effect of temperature?

The Villarreal paper is cited in the S2 Table (and its data was used to build the corresponding Fig2). Since it was not the only paper that studied the impact of temperature, we chose to cite the table instead of the single reference. For example, line 108-110: “Overall, wingbeat frequency increases with temperature, and at high temperatures, wingbeat frequency also increases with humidity (S2 Table).”

NEW COMMENTS

14) “There was a strong variability in willingness to fly between individuals, with some mosquitoes flying non-stop throughout the 10min period, and some flying only for a second when stimulated. We included all mosquitoes that were observed flying in the analysis, regardless of their flight duration during the trial.”

—> If available, it could be interesting to have statistics on the duration of fly, and whether it is species specific, to check if circadium-dependant factors can affect this, e.g. whether night species only had short flights etc

We agree that investigating aspects of flight duration in more details would be an extremely interesting direction. However, in our design there is no robust way to assess flight duration in a standard way in each individual. Since our primary focus in this study was on characterizing mosquito vocalization, we applied a methodology that makes sound records. Based on such approach, we could extract info on flight duration for only those individuals that were flying close enough to the microphone. It is possible that the mosquito sound was not always detected by the microphone even when the mosquito was visibly flying, and this probability depended on the current position of the individual in the cage and the amplitude of its sound. Therefore, for the appropriate characterization of song duration we would have needed an approach that relies on video recordings as well in parallel to the sound recordings — which was initially not considered given the focus of the study. Therefore, we suggest not to present statistics on flight duration as assessed from the sound recordings, because this would represent a biased sample of individuals.

15) the cage is 10x10x10 cm3. Could this small dimension have an effect on the mosquito flight? Could it be a limitation of the study as compared to natural conditions?

While for small and medium species the cage appeared big enough to allow normal flying, it is true that for bigger species, the cage could affect their flight. However, this cage size allowed for the best trade-off between picking up the mosquito sound for small and quiet species, and allowing enough space for mosquitoes to fly naturally. This limitation was added into the manuscript, lines 192-196.
